# Graduate Study in America

**Published:** 1909-05-08

**Authors:** 


### HOSPITAL ADMINISTRATION.

### CONSTRUCTION AND ECONOMICS.

### GRADUATE STUDY IN AMERICA.

THE NEW YORK POST-GRADUATE MEDICAL SCHOOL AND HOSPITAL—(*concluded from p. 114*).

A few days' attendance at the lectures, clinics, and demonstrations given at the school convinces the visitor that the institution is almost ideal of its kind. The teachers have grasped—more thoroughly and more sympathetically in our opinion than is the case at Milan, Vienna, or Moscow, and certainly every whit as whole-heartedly as in the excellent classes at Berlin, Cologne, and Dusseldorf—the fact that graduate instruction, to be efficient, must differ from the kind of instruction that is given to unqualified students. We find, accordingly, that the specialists who are lecturing or demonstrating here give the men hints and practical notions which are not mere book-maxims. Students are taught more by example than by precept, owing to the material available for demonstration purposes, to become familiar with treatment in all its branches, with up-to-date diagnostic methods, and with operative procedure and after-care. The first demonstration which we had the pleasure of attending proved this in an interesting fashion. This was a demonstration by Professor Caillé on diseases of children. A variety of cases was demonstrated, ranging from a simple capillary bronchitis to a meningitis; those attending were asked to examine each patient and confirm the diagnosis. Questions were freely asked and answered, and at the end a most lucid and informative exposition of substitute feeding was given, it being intimated that upstairs, in the wards, the nurse in charge would be delighted to give anyone a practical demonstration in preparing mucilage, acidulated milk, or any of the dozen substitute foods which had been discussed. Similarly at the operations the steps of each were gone over, the reasons for each individual one explained, and the anatomy of the region briefly elucidated by the professor. It was satisfactory to note that in nearly every case the most modern principles were enunciated, while here and there special work was alluded to. This was specially noticeable in Professor de Garmo's interesting clinical lectures on hernia, and Professor Lloyd's no less interesting demonstrations on empyema. The men attending the demonstrations are graduates from all parts of North America—about 600 matriculating each year. They come for varying periods, and either take a special or the general course or stay for a longer time and take advantage of the unrivalled opportunities the hospital affords, by its house appointments, to acquire a more extended experience in one or other branch which specially interests them. They are keen, eager, willing to work, with active interest in their work and its possibilities. The staff, as we have already mentioned, is fully representative, including such well-known names, besides those already mentioned, as those of Drs. Emmet, Porter, Quintard, Rice, Hammond, Boldt, Meyer, Guiteras, Morris, Einhorn, Gant, Taylor, Fuller, McGrath, Cabot, Knopf (who occupies the special chair of phthisiotherapy), Torek, Lusk, and many others. Each member is attached to some well-known city hospital and is a recognised master in his subject, and, what is much more striking, each appears to have specially qualified for graduate instruction. The various classes we had the pleasure of attending were all, without exception, characteristically admirable, bright, brief, brotherly, as such demonstrations ought to be. In saying that they fully come up to the best German classes, and that some of them remind one, in their brightness and practical excellence, of those "never-to-be-forgotten-by-those-who-have-attended-them" talks which made Sir Henry Littlejohn one of the most popular of Edinburgh teachers, we are paying the school and everyone concerned in its work the highest possible compliment.

It would be beyond the compass of our limited space to give anything like an adequate outline of the daily scheme of work at the school. A few points must suffice. There are classes in every specialty, arranged to suit the convenience of students. Practical classes are of strictly limited membership, and this valuable rule is never broken. Men attending the surgical, gynæcological, eye, nose, throat, and ear classes are actually the assistants of the professors; every opportunity is given for practical and theoretical work both in the laboratory and at the bedside. Private coaching classes are given by arrangement, and as a matter of fact, by consultation with the secretary any matriculate can secure demonstrations in any speciality he desires. The ordinary courses, again, are thoroughly sound. Let us take, for example, that in surgery as scheduled. Courses in operative surgery are divided into two sections, operations on the living subject and on the cadaver. The former is again subdivided into Class A and Class B. Each class under Section A, which may be taken as a type, is limited to graduates who work under an adjunct professor, and do a certain number of operations on hospital patients, one of them dressing alternately. Each one does eight operations, which, as scheduled, must include four major ones, such as abdominal, cranial, or larger thoracic. The fee is a hundred dollars, all instruments being provided. Similar courses are given in major gynæcological operations, hernia operations, and operations on the eye, ear, nose and throat, and in orthopædic surgery, etc. Courses on the cadaver, limited to four participants, at fees varying from 25 to 50 dollars, are given in the winter session, and are thorough and full. Regional anatomy courses are given by Professor McGrath for those who desire to supplement their operative work by a course in special dissections. Special classes in particular subjects, such as endoscopy and cystoscopy, are provided, and have proved very popular. In addition there is a fine large reading-room, and, although the club part of the institution has been much less developed than it has been, for instance, at the London Polyclinic, it is the intention of the directors to look after this branch of the institution in an adequate manner when the plans for the alterations are finally revised.

The work in the lying-in department is steadily increasing, and the hospital affords excellent opportunities for those who desire to specialise in gynæcological subjects. There are several women's wards which are always well occupied, and there has recently been developed a special "maternity district," which is in charge of the hospital *internes*, and grants them, for a period of several months, unrivalled opportunities for private obstetric work. A special part of the city, in the neighbourhood of the hospital, is the "Post-Graduate District"; it is visited by the students of the institution, who have been doing praiseworthy work in this section among the poor who cannot afford visiting physicians' fees. In this manner the hospital replaces our district dispensary, such as that of Surrey or Marylebone, and for this work it receives the small city grant already alluded to.

The school is open only to those who have a legal right to practise in the country or State from which they come. There is no distinction made between male and female graduates, with the exception of a few tutorial classes limited to lady graduates. A general ticket available for a year, and admitting to all lectures, demonstrations, and classes except those specially scheduled, costs 450 dollars, a six-weeks ticket 100 dollars, while intermediate tickets can be purchased at proportionate prices. A summer term ticket costs 150 dollars, available from June to October, a monthly term ticket 45 dollars. These fees may appear high in comparison with those exacted in Germany or Italy; but when the difference in price of living and in the general value of money is borne in mind, they must be regarded as being really reasonable. Special coupon tickets are issued to resident physicians in the city who wish to avail themselves of the classes in leisure hours, and these, we understand, are exceedingly popular.

We have laid stress on the admirable opportunities which the institution affords for practical work. In accordance with the prevailing American rule, the four vacancies on the house staff are annually filled by graduates chosen by competitive examination, a system which has worked well so far. These *internes* hold office for two years, during which time they have the best chances for perfecting themselves in their work. There are also ten *externe* vacancies, which are filled quarterly, and for *internes* in special departments, which are filled yearly. All these chances make the institution one of the best for the purposes of the graduate student who has the time and the energy to devote a couple of years to special study. Nor, from what we find, does the institution hold men back from being true to the ideal of graduate study in its best sense by travelling abroad to study other methods and perfect their knowledge by comparison. Many of its students cannot afford to do so; but a number go to Europe, while others again return to the school for a course every year or after a longer period of private practice. The influence of the school has been for good all along the line. It has been the pioneer institution in America, and it has done admirable work—work that can hardly be sufficiently appreciated—in interesting the profession in the graduate instruction ideal. It would be invidious to compare it with other institutions which serve a similar object; in a manner it would be impossible to do so, for no other institution stands in exactly the same position.

This, however, we may be allowed to say in conclusion. The New York Post-Graduate Hospital and Medical School stands as the realisation of an ideal which many members of the profession in England cherish, and which some of us hope to see carried out in the near future. There have been great difficulties in realising that ideal in America; there are greater difficulties, perhaps, with us. Here the inequality of medical qualifications has hampered the institution to a certain extent; in England the graduate study ideal has hardly yet reached that state of development which makes a national effort possible to carry out the aspirations of its enthusiastic supporters. Recently, however, the proposal has been made to devote the munificent legacy to hospital interests made by the late Mr. Barnato to the founding and endowment of a graduate hospital. If that proposal is carried into effect, the executors cannot do better than take into serious consideration the work and the excellent progress which the New York institution have made. For the Post-Graduate Hospital and Medical School may well stand as a model and pattern for any other institution which is to be founded on similar lines, since it stands to-day as a practical illustration of the possibility of combining a graduate instruction school with a general hospital for mutual benefit and gain.

In conclusion, we desire to express our thanks to Dr. Brush and the various professors and demonstrators of the hospital for their unfailing courtesy and help, without which it would have been impossible to investigate the institution and report upon its progress.

